# Fulfilment of the promises of 5G according to business and industry stakeholders in Europe and the United Kingdom

**DOI:** 10.1038/s41598-025-26376-4

**Published:** 2025-11-27

**Authors:** Paige M. Hulls, Gemma Castaño-Vinyals, Martin Röösli, Wout Joseph, Kinga Polańska, Piotr Politański, Mònica Guxens, Frank de Vocht

**Affiliations:** 1 Population Health Sciences, Bristol Medical School, University of Bristol, Canynge Hall, 39 Whatley Road, Bristol, BS8 2PS UK; 2 ISGlobal, 08036 Barcelona, Spain; 3 Universitat Pompeu Fabra, Barcelona, Spain; 4 Spanish Consortium for Research On Epidemiology and Public Health (CIBERESP), Instituto de Salud Carlos III, Madrid, Spain; 5 IMIM (Hospital del Mar Medical Research Institute), Barcelona, Spain; 6 Swiss Tropical and Public Health Institute, 4123 Allschwil, Switzerland; 7 University of Basel, 4003 Basel, Switzerland; 8 Department of Information Technology, Ghent University\IMEC, 9000 Ghent, Belgium; 9 Department of Environmental and Occupational Health Hazards, Nofer Institute of Occupational Medicine, Lodz, Poland; 10 Department of Electromagnetic Hazards, Nofer Institute of Occupational Medicine, Lodz, Poland; 11 Department of Child and Adolescent Psychiatry/Psychology, Erasmus MC, University Medical Centre, Rotterdam, The Netherlands; 12 National Institute for Health Research Applied Research Collaboration West (NIHR ARC West), Bristol, UK

**Keywords:** 5G, Electromagnetic fields, Workplace, Employees, Electrical and electronic engineering, Risk factors, Environmental economics

## Abstract

For businesses there is now the opportunity to incorporate fifth generation cellular technology (5G) into working practices to, for example, deliver contextual information in real time by ultra short latency and connect large numbers of devices, alongside artificial intelligence applications. The aim of this study was to gain insights into the development, implementation and the use of 5G in Europe, and to obtain an overview of developments associated specifically for occupational settings. We interviewed 14 experts from business, industry and (inter)national stakeholder organisations from the UK, Belgium, Spain, Poland and Switzerland between March and September 2023 using a semi-structured topic guide. Interviews were then transcribed, coded and analysed. Participants had mixed opinions about 5G as “*it will not be the solution to the problems that we thought*”, but “*5G is still developing*”. The introduction of 5G in workplaces was viewed as *“pretty small”* and in several countries *“hasn’t been very well taken up”*. Occupational settings where 5G had been initially incorporated included farming, manufacturing, airports, and university campuses. Introduction was also influenced by government-funded schemes to pilot 5G within businesses. Participants felt 5G could lead to a *“natural evolution in factories”*. However, this would require continuing investment in user equipment and resources, as *“all equipment would need to be able to connect to the 5G network”*. Experiences varied, but participants acknowledged that COVID-19 had had an impact on public perception, with misinformation being a prominent factor. Employment of 5G in industrial settings in Europe has been behind expectations, and to date mostly limited to test sites. Although work began in 2015, 5G deployment is continuing across Europe. Further research is needed to understand how businesses can effectively implement 5G.

**Supplementary Information:**

The online version contains supplementary material available at 10.1038/s41598-025-26376-4.

## Introduction

‘5G’ refers to the fifth generation of broadband data-networking digital communication^1^. 5G follows on from 1G in the 1980s, 2G, the first digital mobile technology, 3G which enabled the launch of smartphones, and 4G, launched in 2012, which allowed users to have a faster browsing experience^2^. The average latency for 4G is around 50 ms, whilst the average of 5G is around half of that time and can even go as low as one millisecond^3^. The requirements of 5G are expected to be met by new spectrum in the microwave bands (3.3 – 4.2 gigahertz (GHz) and 700 megahertz (MHz)) and utilising large bandwidths available in mm-wave bands^4^. Findings from the Special Eurobarometer 2023^5^ reported that 4 out of 5 general population respondents consider that digital technologies will be important in their lives by 2030, and recognised the need for stronger cybersecurity, better connectivity and better protection of data (3 out of 4 respondents). The three main priorities identified by European respondents were: protecting users from cyberattacks, improving the availability of high-speed internet for everyone and everywhere, and protecting users from disinformation and illegal content. The Digital Decade Policy Programme discusses the EU’s objectives to ensure uninterrupted 5G coverage in urban areas and along main transport paths by 2030. A total of 81% of overall 5G coverage has been achieved across Europe, as published in the first ‘Report on the state of the Digital Decade’ in September 2023^6^. The UK Government has described 5G technology as offering three main differences in comparison to 4G: faster data speeds, high capacity and faster responsiveness/low latency^7^. The UK government’s roll-out strategy was publicised in the 2018 Future Telecoms Infrastructure review^8^, with ‘all populated areas’, including rural communities, to have standalone 5G coverage by 2030. So far, urban areas have been prioritised, and in the UK between 85 and 93% of premises have 5G available outdoors^9^.

Alongside benefits to the consumer and households, there are also significant advantages to businesses incorporating 5G into their practices. A report by Barclays reported that the introduction of a 5G mobile telecommunications network could increase aggregate UK business revenues (in 2018 prices) by between £8.3 and £17.5 billion by 2025^10^. For the European economy, 5G should add up to 1.0 trillion to the European Gross Domestic Product (GDP) between 2021 and 2025 as well as potentially creating or transforming 20 million jobs across all sectors^11^.

5G has the potential to enable various industrial ‘smart applications’, particularly in health and social care, transport and logistics (including ports), manufacturing and agriculture^12^. VR/AR, ultra-high definition (UHD) video, robotics, autonomous/connected vehicles, drones, remote object/machine manipulation and smart tracking are examples of existing applications that can be enhanced by 5G^7^. The Worcestershire 5G Testbed, a UK government-backed trial reported that the use of augmented reality technologies indicated a potential productivity benefit of 2%^13^.

The impact of 5G is, however, difficult to measure because it is dependent on engagement and uptake. 5G uptake is lower in European countries than North America, China, Japan, and South Korea. Although in 2024 most European countries (34 out of 50) had deployed 5G and over half of operators had launched 5G networks (92 out of 173)^14^, some, such as for example Poland, do not yet have a dedicated 5G spectrum (as of 2023). In December 2023, the rollout of standalone 5G in the UK was ‘at an early stage’; around 2,000 sites deployed, out of an anticipated 18,500 deployments in total^9^. For the UK, the delay was partially attributed to the government’s order to remove China’s Huawei equipment and services from core 5G network functions by 2027^15^. The deployment of 5G requires significant investment and whilst there are clear potential advantages to consumers and businesses to engage with 5G, there is continued uncertainty around uptake and adoption^12^. Wider challenges include practical deployment barriers (planning, regulation and site landlords), public opposition to 5G relating to health concerns, security concerns regarding national infrastructure and access to sufficient spectrum for mobile operators to offer 5G services^12^.

This study is part of a wider five-year programme commissioned by the European Union’s Horizon Europe Research and Innovation Programme, GOLIAT, aimed at providing responses to some of the questions raised by the new wireless technologies, with a special focus in 5G^16^.

The aim of this particular study is to gain insights from European stakeholders about the development, implementation and/or use of 5G technology in Europe. We primarily focus on exploring opinions on the main trends and developments associated with 5G exposures in occupational settings, among stakeholders and professionals from the various industries. Secondarily, we explore how this is influenced by perceptions around 5G amongst the general public. Thirdly, we explore perspectives with regards to where and how 5G technologies will likely be implemented in the near future.

To the best of our knowledge, this is one of the first qualitative studies to explore the trends of 5G rollout in the UK and across Europe, as well as the implementation of 5G in the workplace.

## Methods

We conducted semi-structured interviews with stakeholders and professionals across various business, industry and (inter)national stakeholder organisations, involved in the development, implementation or use of 5G and/or associated technologies from the UK, Belgium, Switzerland, Poland, and Spain. Participants could be located globally provided that their remit covered Europe and/or the United Kingdom. Anonymised summary findings were made available to participants, but they (or the companies they are employed by) played no role in the study design, analysis, and interpretation of findings.

A recruitment email was sent to the pre-identified experts and stakeholders, outlining the research and asking them to contact the lead researcher (Paige M Hulls (PMH)) if they wished to take part. Participants were pre-identified by the research team as part of the wider project’s engagement between June and December 2022. Participation was voluntary. Interested participants were sent an information sheet and consent form and subsequently contacted by PMH to provide some basic information and arrange an interview time. Interviews were conducted via Microsoft Teams between March and August 2023 by PMH and were audio recorded. A total of 13 individuals did not respond to the initial invitation and follow-up invitation email, whilst six participants declined to participate after initially expressing interest; no reasons were given. Participants provided written informed consent before starting the interview.

We used a semi-structured topic guide (Appendix 1) developed by PMH and FDV to ensure key topics of interest were covered, while allowing participants to raise new issues. An initial draft topic guide was sent to all co-authors and comments, suggestions, changes and additions were incorporated until all researchers approved the final topic guide. Interviews focused on the following topics: participant background and experience; the use of 5G and 5G in occupational settings; and the future of 5G technologies in industry and occupational implementations. Participants were offered the opportunity of having a native speaking researcher present in the interview if English was not their first language, however no participants requested this. Interviews ranged from 25 – 50 min with additional field notes made after each interview.

Interview recordings were transcribed, anonymised, and checked for accuracy. To maintain participant confidentiality, names of countries were removed. PMH read the transcripts to be able to familiarise with the data and note potential codes using an inductive coding process. These were developed into a coding framework, which was subsequently applied to further transcripts by PMH. If new topics occurred during coding of interviews these were added and retrospectively coded to earlier transcripts. NVivo 12 was used to facilitate data management and analysis^17^. In this exploratory study double-coding was not conducted. However, FDV reviewed the topic development process and final topics agreed after several iterations. FDV reviewed the coding of the interviews by PMH, and differences were discussed and coding agreed. We present illustrative quotes to support our analysis.

Ethics approval for this study was granted by University of Bristol’s Health Sciences Faculty Research Ethics Committee (ref 12,524).

## Results

We interviewed 14 professionals (12 males, 2 females): 7 from the UK, 2 from Spain, 1 from Switzerland, 2 from Belgium and 2 from Poland, as presented in Table 1. They were involved international telecommunications companies management, RF health and safety system engineering, researchers in aspects of telecommunications, independent EMF consultancy, and government officials responsible for radiation-related injury prevention, spectrum planning and safety, and national 5G implementation strategy. Three themes are presented following the analysis of the data: overview of the main 5G trends, 5G in the workplace, and the future of 5G.

**Table 1:** Participant characteristics.

Participant country	Number of interviews	Participant gender	Employment sector	Years of experience
UK	7	Males: 5 Females: 2	Government: 3 Private: 4	10–19 years: 1 20–29 years: 3 30–39 years: 1 40–49 years: 2
Spain	2	Males: 2	Government: 2	10–19 years: 1 30–39 years: 1
Switzerland	1	Male: 1	Private: 1	10–19 years: 1
Belgium	2	Males: 2	Government: 1 Private: 1	10–19 years: 2
Poland	2	Males: 2	Government: 1 Private: 1	20–29 years: 2

### Introduction of 5G

Participants identified that in the majority of instances, 5G is using the 3.5GHz – 3.6GHz band. In the UK, participants noted that *“operators have been re-farming some of the existing spectrums”* [Identification participant 8 (IDP8)], where 2G network has already been switched off and 3G is due to be switched off. Across Europe, the 3.5GHz – 3.6GHz band has been identified as *“exclusively for 5G at the moment…that operators could buy a license for”* [IDG23]. In Poland, mobile operators *“have started 5G with the legacy bands based on 2.1GHz, as well as an additional spectrum for TDD services at 2.6GHz”* however *“the only thing we lack is the spectrum…but we still hope it will happen this year (2023)”* [IDG18]. After data collection had ended and at the end of 2023, the auction for the C band was resolved in Poland.

Participants commented that in their opinion, 5G had not been deployed across Europe as quickly as initially anticipated. Several factors were identified including the capacity of the networks as well as the cost of the infrastructure needed: *“Then, you’ve got the reality of actually having to pay for the infrastructure to do all that and it might have the bandwidth to do it all, but you’ve still got to have the kit to do it all”* [IDP10].

Participants discussed the impact of launching 5G and the COVID-19 pandemic in the same timeframe: “*So you’d got these two things happening that people were blaming COVID on 5G and there was all that, the sites being set on fire in 2020. It was absolutely horrendous, a whole load of people getting all sorts of abuse”* [IDP10]. Participants who were involved in working on the deployment of 5G across Europe also found that *“COVID delayed a lot of technical deployment and delayed a lot of equipment availability. And in the 5G ecosystem, when I was working in* < *country* > *, particularly there wasn’t a lot of choice of vendors. Whereas now, there’s a much bigger market, the price point’s come down”* [IDG31]. In the UK *“it definitely slowed down delivery, it slowed down deployment and slowed down the ability”* [IDG32].

### Perceptions of 5G among the general public

The interviewed business and industry stakeholders recognised that there was an assumption among the general public that higher frequencies are being used for 5G deployment, and which the general public perceived as being harmful to the health of users. *“In the* < *country* > *, we don’t even have the 5G on those higher frequencies, on 28 GHz or whatever, but I don’t think a lot of the public understand enough to understand that distinction”* [IDP10]. Particularly since the launch of 5G, there was greater concern about the construction of base stations and the location, *“not realising that they need the base station to make the call to complain”* [IDG12]. However, they acknowledged that concerns about health is *“a trend with any technology we can see…about 10 years ago we had similar sort of concern about Wi-Fi”* [IDG14].

Participants also discussed that how we use phones has changed since the launch of 2G which can impact the user’s exposure, although this is not necessarily well known and/or recognised among the general public.

*“Most of your exposure comes from holding a source close to your body or close to your head. Of course, the modern way of using a phone is more like having it in front of you like that (participant gestures). And because as the technologies go forward, there’s, kind of, good engineering rationale for not transmitting more signal than you have to” [IDG12].*

Professionals explored the different approaches that various agencies across the UK and Europe took by either issuing statements regarding 5G and the COVID-19 pandemic, following the World Health Organisation or *“we didn’t feel we wanted to give it credence by producing a statement on it”* [IDG12]. Many felt that the increased misinformation and conspiracy theories, particularly via social media, Which the burning of mobile communications towers was attributed, made working conditions more challenging:

*“At the time, we had to disable our map with the location of all the antennas so that the activists couldn’t find them”* [IDG23].

### 5G in the workplace

Participants were in agreement that for businesses that are technologically advanced and/or with real-time data stream requirements *“having such a technology is a benefit, no question at all”* [IDG30], as well as the wider benefits relating to *“the security compared to the traditional Wi-Fi solutions”* [IDG30], particularly for managing and/or transferring confidential data. However, they felt that there was a disconnection between what 5G could provide when deployed compared to what is currently being offered to businesses and their workforces, alongside the capacity available. *“This massive use that was foreseen in terms of having the industries with plenty of 5G deployments or having some of the hospitals also with plenty of 5G deployment, for having some kind of telesurgery *etc*., this is far from being a reality. Very far”* [IDG30].

Initial business interest in 5G was not yet widespread and still dependent on companies recognising the potential of *“a business model in 5G because it’s a specific technology with a lot more advantages over 4G"* [IDG23]. In several countries, 5G has been deployed for businesses, however businesses have not engaged with 5G as quickly as initially thought: *“* < *country* > *has some dedicated spectrum, but my understanding is that it hasn’t been very well taken up. So they have much less demand than they expected”* [IDP15].

One of the biggest barriers for companies was the financial investment required. *“I think that the big hurdle which needs to be overcome is still the pricing, apart from the health concerns and the other risks involved. I think, definitely with the economic crisis, which is happening now, the energy prices which are going up, making investments is something which is now difficult for companies. So for instance, changing of private 4G network to a 5G network, well, you need to really be sure that it’s beneficial for your company”* [IDP28]. Participants felt that the economic crisis and workflow security were factors associated with companies postponing investments in technological upgrades: *“They want to make sure that people working there still have work in two years”* [IDP28], as well as the need for upgrading existing software, *“the user equipment, is, most of the times, not 5G compatible yet. So that’s a big problem”* [IDP28]. Government grants had been offered in several European countries to promote the deployment of 5G in businesses in “pilot-type scale” [IDP15], which had *“helped a lot of companies to invite in the private 5G networks”* [IDP28], and in turn, further applications were being made as a result of successful grants.

Participants recognised that during the deployment of 5G private networks within businesses, there could be potential discourse between employees and those living in the surrounding areas – *“So I think there shouldn’t be problems with giving the information to the employees. Like, “In this factory, in this area, there is a 5G network that is controlling everything,” like drones or some autonomic vehicles and everything. This could be a problem for the neighbours, for example, or for those living nearby”* [IDG18].

Participants identified several industries, including manufacturing, farming, ports *(“5G is quite a big thing in ports”* [IDG32] and airports (*“you can’t put Wi-Fi nodes all down the runway”* [IDP15]) where 5G is being deployed. Automating processes within factory settings was identified as an area that could benefit from 5G, and potentially providing businesses with clear financial reward: *“It’s a great solution to automate a lot of processes. When you have a lot of repetitions in a process and a lot of automation, then 5G is really a great solution for that. It seems like a natural evolution in factories”* [ID18]. Participants were also receiving interest from educational sectors, *“campuses, universities, schools and places where people use information a lot, or need access to information fast. I think this also has a very bright future in those places”* [IDG18].

The deployment of 5G in conjunction with emergency services was discussed multiple times, and the importance of providing 5G coverage where *“they can’t get it from terrestrial coverage they can get full coverage from satellite 5G”* [IDG14]. An example of where 5G deployment would have been advantageous was during the 2019/2020 Australian bushfires where *“there were incompatibilities between the radios used by firefighters in one state and firefighters in the adjacent state”* [IDP15]. It was felt that the use of 5G as part of ‘mission critical applications’ is “*where it has been a key player or where it is driving change”* [ID28]. Government grants had enabled the use of drones across every fire department in one European country, so the scope of the fire can be determined before crews arrived on site – *“we call that beyond visual line of sight flight”* [IDG18].

### The future of 5G in businesses

For the general population living in more populated areas, it was generally accepted that *“5G is an integral part of our life now”* [IDG14]. However, they acknowledged that there continued to be challenges in more rural areas and/or cities where there is high architectural importance: *“The local authorities usually object to an erection of a mast that would change the scenery, especially in old cities like* < *country* > *which has certain characteristics”* [IDG14].

Several participants spoke about the difference in what 5G was expected to offer versus what is being offered – *“5G will not be the solution to the problems that we thought five years ago. That’s the reality”* [IDG31]. At the time of conducting the interviews, 5G deployment varied across European countries, and therefore, capacity was inconsistent for both the general public—*“The problem is, in* < *country* > *, we still can’t get the real experience, because of the lack of spectrum”* [IDG18] and businesses – *“In industry, here in* < *country* > *, there is no possibility now, I think that in the next future it will be possible, to use frequency to create private networks on 5G. In the* < *country* > *, there is a spectrum in order to implement it, but not here”* [IDG31].

Within businesses, although 5G could lead to a *“natural evolution in factories”* [IDG18], participants felt that it depended on the businesses themselves to determine whether their level of engagement with 5G: *“It’s a matter of maturity of the corporative potential customers, interpreting 5G as a technology that is going – it might help them in solving some of the traditional problems that they are facing with non-5G networks”* [IDG30].

### The future of 5G among the general population

Participants recognised that the uptake of 5G was dependent on how users and the general public engaged with 5G on their smart devices – *“once you can stream your film without buffering or whatever, what is it that you can’t do that you’d need the technology to do or the 5G to do? If you can already do everything you want, it gets very difficult to actually improve…”* [IDP10]. The frequency of which people are upgrading their mobile phone has also changed in the last decade and participants felt this was due to the financial cost and environmental pressures: *“Then, every year, you’d get a new phone. So, there was a, sort of, status about having new phones all the time. Now, I notice there are a lot more adverts for SIM-only contracts, where you don’t have to upgrade. From the environmental perspective, it’s a lot more socially acceptable, I think, to not have the fanciest phone”* [IDP10]. It was also noted that inevitably the conversation will shift onto 6G and this may cause a reluctance to engage with 5G—*“Oh, well, 4G was a big thing and now we’ve got 5G. They’re already talking about 6G. So, let’s not bother committing to the 5G, let’s go straight to the 6G”* [IDP10].

It was clear that as 5G deployment continues and particularly in the wake of the COVID-19 pandemic, participants recognised the importance for public engagement to address health concerns and misinformation, *“once you lose public opinion, it’s very difficult to get it back”* [IDP7]. Examples of good practice regarding the engagement of companies with 5G users were explored and the benefits of doing so: “*We teach the people what 5G is, what the health risks are involved, if there are any. What’s the difference between 4G, what’s the difference between Wi-Fi, and we also talk about the regulations that are in place by Europe…so we try to inform the people as much as possible that it’s actually safe”* [IDG28]. However, there appears to be a lack of clarity with respect to who is responsible for delivering this type of information – government, operators or companies using 5G. Lack of engagement led to *“a lot of anxiety with the people and also a lot of applications for measurements in their homes”* [IDG23]. Despite the existing literature regarding 3G and 4G, working around 5G and addressing public concerns continued to be a challenge.

*“It’s a double-edged sword, because if you don’t do research, you are not doing anything. If you do research, “Oh there must be something wrong. There must be something dangerous about this technology that you are doing research”* [IDG14]*.*

## Discussion

The aim of this study was to gain insights into the development and/or the use of 5G in Europe and the United Kingdom and obtain an overview of developments associated specifically for occupational settings. Interviews with participants from across various business, industry and (inter)national stakeholder organisations, had mixed opinions about 5G. The introduction of 5G in workplaces was viewed as *“pretty small”* and as of 2023, in several countries *“hasn’t been very well taken up”*. Participants felt 5G could lead to a *“natural evolution in factories”*. However, this would require continuing investment in user equipment and resources, as *“all equipment would need to be able to connect to the 5G network”*. Experiences varied, but participants acknowledged that COVID-19 had had an impact on public perception, with misinformation being a prominent factor.

Incorporation of 5G in businesses varied across countries and industries. The majority of participants identified airports, ports and university campuses as commonplace locations where 5G had been implemented, whilst in Belgium, 5G providers were engaging with emergency services. Examples included trialling the use of drones to provide a rapid assessment to dispatching crews^18^ and development of a 5G radio network to improve the communication system between the emergency telephone number and dispatching centres^19^. Airports have been previously identified as a setting where dedicated 5G networks could be beneficial, both for travellers and operations^20^. In the UK, five airports now have 5G networks accessible to travellers—Belfast, Birmingham, Newcastle, Edinburgh and Glasgow^21^. Whilst not commonly identified in our interviews, the integration of 5G into the healthcare sector has been well-documented, with practices focusing on remote patient monitoring, robotic surgery and data repository^22^.

Participants discussed the financial investment for new equipment and resources that would be needed to support 5G technology. This has been highlighted by others “*given that 5G hardware will be more expensive than that of 4G*”^23^, alongside recognising the challenges of measuring the value of 5G use. The Digital Connectivity Forum’s report found that it is likely there could be a £3 to £5 billion investment gap for the deployment of basic 5G functionalities in the UK and therefore, the UK wide rollout of 5G may not be achieved by 2030^24^. Kiesel, et al., (2020) described the lack of detailed approaches and models for evaluating the application of 5G technology in production^25^. A lack of certainty regarding the monetary benefits has been previously defined as a key concern for businesses and therefore systems or approaches need to be in place to ensure that production processes are able to be quantified^25^. Businesses will also need to recognise that investment will depend on the local demand and cost conditions in the areas in which they are deployed, e.g., urban versus rural areas^24^.

Although several reasons were identified due to the possible lower than expected rollout of 5G, participants noted the potential impact of the COVID-19 pandemic and misinformation surrounding 5G. COVID-19 was first identified in Wuhan, China, in December 2019, weeks after the city-wide 5G became operational on 31^st^ October 2019^26^. Previous papers have reported that established conspiracy theories suggested there was a causal relationship between the spatial distribution of the density of COVID-19 infection and the density of 5G towers^27–29^. This theory was shared by several media personalities including Eamonn Holmes, Amir Khan, John Cusack, Woody Harrelson, and MIA^27,30^. Participants highlighted that companies were consequently more reluctant to openly promote the implementation of 5G in their workplaces, due to concerns about public backlash and equipment and/or infrastructure damage. At least 77 mobile towers in the UK were burned, as a result of conspiracy theories^27,31^. In Europe, the Netherlands had 16 arson attacks on 5G mobile masts, Ireland had three arson attacks, and Belgium, Italy, Cyprus and Sweden have had at least one^32^.

To the best of our knowledge, this is one of the first qualitative studies to explore the trends of 5G rollout in the UK and across Europe, as well as the implementation of 5G in the workplace. We were also able to engage with participants with a range of occupations to gain a wider view in several European countries and allow comparisons to be made. This qualitative study was not aimed at representativity, but aimed to explore the most common arguments and opinions amongst stakeholders. However, we acknowledge that the sample size of this exploratory study is smaller than anticipated (n = 14), and included only two women, and therefore the views expressed may not be wholly representative. Moreover, as half of our sample were based in the UK, views may heavily reflect the perspective from this country. Nonetheless, their views did not materially differ from those from the other European countries. Lower than expected recruitment within this study has also been experienced in other studies recruiting businesses and workers, as part of the wider GOLIAT project. Given the variety of sectors from which we drew our sample, and views being generally congruent, we believe that despite the smaller than expected sample the current exploratory work is representative of the common arguments and opinions in the wider community. Furthermore, participants were recruited through researcher connections and other relevant stakeholders may not have been aware of this piece of research. To obtain the high level experience from national and international stakeholders, we focussed recruitment to government and large, international, organisations. However, the implication of this choice is that the views and experiences of small and medium sized enterprises, workers, and specific end-user groups were underrepresented. This is an important gap as their experiences may differ from those of international stakeholders and governments.

The opinions of the stakeholders in this exploratory study reflect experiences during the first years of the implementation of a new technology, and the general opinion of stakeholders was that development had been behind expectations. Although implementation of 5G in the general population has continued with about 89% of the European population having access to 5G mid-2023 and 90–95% of UK premises in 2024, the 5G pioneer bands (700 MHz, 3.6 GHz, and 26 GHz) have not been completed (and should have been by 2021 completed across the EU). Industrial implementation has been underdeveloped and remains restricted to 5G trials, particularly in ports, and standalone 5G networks implementation remains behind expectations^33,34^.

Given the complexity of the topic that has societal, environmental, engineering, and other impacts it is essential that future research taken an interdisciplinary approach. Future research needs (and in coming years, this may change) to prioritise engaging with businesses, to understand their decision-making processes, and to explore why uptake has been lower than expected. Businesses should have access to clear cost–benefit models to help them fully understand the financial investment required and possible return, alongside the identification of any relevant financial support. Sector-specific detailed analyses would further benefit these evaluations, and our results will help any future such research to develop a targeted questionnaire for quantitative research. Future, more comprehensive, research would further benefit from inclusion of workers. Researchers should engage with employees working in 5G-implemented areas to ensure that any health concerns they may have regarding 5G exposure can be addressed.

## Supplementary Information

Supplementary Information.

## Data Availability

The data that support the findings of this study are available on request from the corresponding author. The data are not publicly available due to privacy or ethical restrictions.
