# Supplementary material for: Fulfilment of the promises of 5G according to business and industry stakeholders in Europe and the United Kingdom
Source: Sci Rep. 2025 Nov 27;15:42383. doi: 10.1038/s41598-025-26376-4 (PMC12660949; doi:10.1038/s41598-025-26376-4)
Supplement: Supplementary file 1 — Supplementary Information. [file 41598_2025_26376_MOESM1_ESM.docx]

**Supplementary Materials**

**Fulfilment of the promises of 5G according to business and industry stakeholders in Europe and the United Kingdom**

Paige M Hulls^1^, Gemma Castaño-Vinyals^2,3,4,5^, Martin Röösli^6,7^, Wout Joseph^8^, Kinga Polańska^9^, Piotr Politański^10^, Mònica Guxens^2,3,4,11^, Frank de Vocht^1,12^

1. Population Health Sciences, Bristol Medical School, University of Bristol, Canynge Hall, 39 Whatley Road, Bristol, UK
2. ISGlobal, 08036 Barcelona, Spain
3. Universitat Pompeu Fabra, Barcelona, Spain
4. Spanish Consortium for Research on Epidemiology and Public Health (CIBERESP), Instituto de Salud Carlos III, Madrid, Spain
5. IMIM (Hospital del Mar Medical Research Institute), Barcelona, Spain
6. Swiss Tropical and Public Health Institute, Allschwil, 4123, Switzerland
7. University of Basel, Basel, 4003, Switzerland
8. Department of Information Technology, Ghent University\IMEC, 9000 Ghent, Belgium
9. Department of Environmental and Occupational Health Hazards, Nofer Institute of Occupational Medicine, Lodz, Poland
10. Department of Electromagnetic Hazards, Nofer Institute of Occupational Medicine, Lodz, Poland
11. Department of Child and Adolescent Psychiatry/Psychology, Erasmus MC, University Medical Centre, Rotterdam, The Netherlands
12. National Institute for Health Research Applied Research Collaboration West (NIHR ARC West), Bristol, UK

**Funding**

This project has received funding from the European Union’s Horizon Europe research and innovation programme under grant agreement No 101057262. Views and opinions expressed are however those of the authors only and do not necessarily reflect those of the European Union or the Health and Digital Executive Agency. Neither the European Union nor the granting authority can be held responsible for them.

FdV is partly supported by the National Institute for Health and Care Research Applied Research Collaboration West (NIHR ARC West).

**Acknowledgements**

We thank all individuals that participated in our study.

APPENDIX 1: PARTICIPANT TOPIC GUIDE

**Introduction**

Thank the participant for taking part, re-introduce myself and re-state the purpose of the interview. Remind them about the digital recording, that they can skip questions if they wish, stop recording or end the interview at any time.

**Participant Background and Experience**

Please can you describe your current job role?

How does your role fit into the business?

What is your occupational background?

How long have you been with this company?

Have you worked with any other companies before your current employer?

**5G exposures in occupational settings**

How would you describe your insights into how 5G is used in your workplace?

Do you have any wider knowledge of how 5G is used in other workplaces and/or industries?

In what capacity do you use 5G in the workplace?

How has the usage of 5G changed during your role?

Are there any other sectors that use 5G in the workplace?

Do you use 5G on the same spectrum as 3-4G (i.e., <3GHz) or higher frequencies up to the millimetre wave frequency?

In your workplace, are there any main trends regarding 5G innovations and applications in occupational settings that you’re aware of?

Are there any potential developments in your workplace that we have not discussed?

**The future of 5G technologies**

If appropriate, where in your workplace do you think 5G could or will be used?

Can you describe your thoughts and/or opinions about incorporating 5G technologies in the workplace in the future?

Do you have any thoughts on where 5G is likely to be implemented in the future?

**Closing the Interview**

Ask the participant if there is anything they would like to discuss or add that has not been covered. Thank the participant for taking part.
